# A Robust and Versatile Mating Function for Two‐Sex Population Projection Models Fitting all Types of Mating Systems

**DOI:** 10.1111/ele.70013

**Published:** 2024-12-02

**Authors:** Jessica Cachelou, Christophe Coste, Jean‐Michel Gaillard, Agathe Chassagneux, Emmanuelle Richard, Eric Baubet, Marlène Gamelon

**Affiliations:** ^1^ Laboratoire de Biométrie et Biologie Evolutive UMR 5558, CNRS, Université Lyon 1 Villeurbanne France; ^2^ Fondation François Sommer Pôle Nature Paris France; ^3^ Office Français de la Biodiversité DRAS‐Service Conservation et Gestion Des espèces à Enjeux Birieux France; ^4^ Department of Biosciences Swansea University Swansea UK

**Keywords:** mating efficiency, mating function, monogamous, operational sex ratio, polyandrous, polygynous, population dynamics, promiscuous

## Abstract

Commonly used two‐sex discrete‐time population projection models rely on mating functions developed for continuous‐time frameworks that overestimate the number of unions between reproductive individuals. This has important consequences for our understanding of the evolution and demography of two‐sex populations and consequently for management and conservation. Here, we propose a novel mating function that is robust by obeying all properties necessary to be ecologically valid and flexible by accommodating all mating systems and efficiency in mating encounters. We illustrate the usefulness of this novel function with an application to the sexually size‐dimorphic and polygynous wild boar (*Sus scrofa*). We show that the population growth rate depends on the harem size, the operational sex ratio, and the mating efficiency. This novel function can be applied to all mating systems and tactics and is highly relevant in the context of global changes under which mating systems and mating efficiency are expected to change.

## Graphical abstract

Commonly used two‐sex discrete‐time population projection models rely on mating functions developed for continuous‐time frameworks that overestimate the number of unions between reproductive individuals. We propose a novel mating function that is robust by obeying all properties necessary to be ecologically valid and flexible by accommodating all mating systems and efficiency in mating encounters. This novel function can be applied to all mating systems and tactics and is highly relevant in the context of global changes where mating systems and mating efficiency are expected to change.

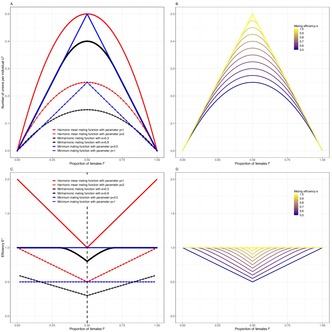

## 1 Introduction

A better understanding of population dynamics is fundamental for management and conservation purposes, especially in the current context of global changes. Most demographic studies published to date have explored population dynamics using demographic models built on females only. They are technically simple and require the monitoring of individuals of that sex only. However, these models rely on the crucial assumption that the number of males in a population has no influence on females' reproduction (and on other vital rates, such as survival). Moreover, they often completely ignore the dynamics of the male component of the population, and when they do not, they often consider that vital rates are identical for both sexes (Pollard 1974; Caswell 2001; Iannelli, Martcheva, and Milner 2005). However, in a large range of species throughout the tree of life, substantial sexual dimorphism occurs in life history traits, causing males to matter (Mysterud, Coulson, and Stenseth 2002). Because male and female mortality trajectories may differ markedly (Pollak 1990; Tidiere et al. 2015; Lemaître et al. 2020; Gamelon et al. 2012) due to, for instance, selective harvesting (Fenberg and Roy 2008; Zhou et al. 2010; Milner‐Gulland et al. 2003), two‐sex models have been developed to account explicitly for sex‐specific vital rates.

The number of males in a population, even when lower than the number of females, may strongly influence females' reproduction (Milner‐Gulland et al. 2003). Therefore, it is crucial to account explicitly for both the number of males and females in a population and to model the number of unions. To do so, two‐sex population projection models *including mating functions* have been proposed (e.g., Legendre et al. 1999; Jenouvrier et al. 2010; Miller and Inouye 2011; Gerber and White 2014; Tahvonen, Kumpula, and Pekkarinen 2014; Tenan et al. 2016; Eberhart‐Phillips et al. 2017). In that case, the number of unions depends on both the number of reproductive males and the number of females that are ready to mate, which define together the operational sex ratio (hereafter OSR; Kvarnemo and Ahnesjo 1996) and on the mating system (e.g., monogamy, polygyny, polyandry, or promiscuity; see Box 1: Glossary for the description of these different mating systems).

BOX 1 **Glossary.**

1 *Monogamy:* a mating system where one male mates with one female in a breeding season.

*Polygyny:* a mating system where males mate with more than one female. For a population, the number of females that mate with one male is defined by the harem size, *h*. In the real world, *h* can vary amongst males within the same population, but a single mean *h* value is considered in the modelling, which corresponds to the mean value at the population level.

*Polyandry*: a mating system where one female mates with more than one male. For a population, the number of males that mate with one female is defined by *h’*. In the real world, *h’* can vary amongst females within the same population, but a single mean *h’* value is considered in the modelling, which corresponds to the mean value at the population level.

*Promiscuity*: a mating system where males mate with several females and females mate with several males.

*Random mating:* each individual has the same probability to mate with another individual.

*Assortative mating:* mating is structured: an individual mates with another individual with the same (i.e., positive assortative mating) or dissimilar (i.e., negative assortative mating) phenotype characteristics compared to itself.

*Efficiency (in mating):* Probability to meet and mate for an individual of the limiting sex. A low efficiency in mating corresponds to a difficulty for individuals to meet and mate. At the opposite, a high efficiency in mating corresponds to easy mating.

**FIGURE 1:**
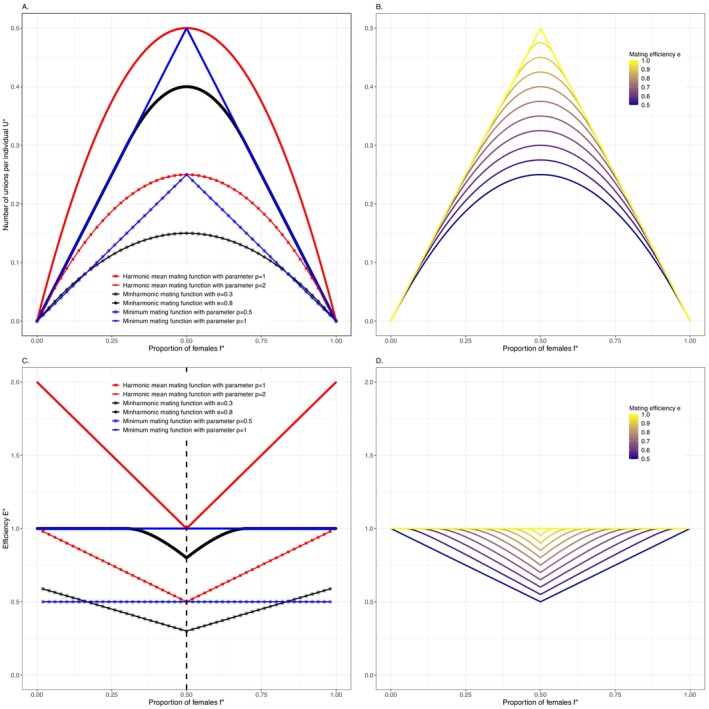
Number of pairs formed $U^{*}$ (A and B) and efficiency $E^{*}$ (C and D) as functions of the OSR (proportion of females $f^{*}$) for the various mating functions described in Box 2 (A and C) and for the minharmonic mating function (Equation (3)) for a range of values of the mating efficiency parameter *e* (B and D).

Different mating functions have been proposed in the literature to account for different mating systems and OSRs within two‐sex models (see Miller and Inouye 2011 for a review of existing mating functions). Theoreticians have introduced a series of mandatory properties that a mating function must satisfy to be mathematically and ecologically valid for continuous‐time models (Fredrickson 1971, Gupta 1972, Yellin and Samuelson 1974; see Supporting Information S1 for a commented list of these rules). However, for population ecologists, discrete‐time projection models are often more appropriate than continuous‐time models, particularly for populations with seasonal or periodic life history, such as those characterised by a short breeding season within a year (Bacaër 2009) or survival rates varying amongst time periods within a year (e.g., for exploited species, Gamelon et al. 2012). This has yielded direct extensions of the continuous‐time functions towards the discrete‐time framework (e.g., Caswell 2001). However, the resulting mating functions, some of them commonly used in two‐sex discrete‐time projection models, fail to meet ecological standards (see Box 2 for the state of the art of existing mating functions). Notably, one of the most heavily used functions, the harmonic mean mating function, even violates the most basic logical property that, in a monogamous framework, “*the number of unions involving a sex must not exceed the total number of individuals of that sex*” (Pollak 1986), which can have severe consequences (see Box 2). More precisely, the harmonic mean mating function creates more pairs than the number of available males or females in the population (see Figure 1A). It is crucial to model accurately and realistically population dynamics using an adequate and robust (to ecological and mathematical validity properties) mating function for discrete‐time models.

BOX 2 **State of the Art on Previously Published Mating Functions in Discrete‐Time Models and Their Main Pitfalls.**

1 Two mating functions are commonly used in discrete‐time two‐sex population projection models: the *minimum mating function* (e.g., Legendre et al. 1999; Jenouvrier et al. 2010; Brodie et al. 2011; Tenan et al. 2016), and the *harmonic mean mating function* (e.g., Caswell 2001, Tsai et al. 2015, Eberhart‐Phillips et al. 2017; see Supporting Information S1).

In two‐sex models, the mating function is, in general, developed for a monogamous population. Here, we show that each monogamous mating function corresponds to a probability for an individual of the limiting sex to meet and mate, which we call “efficiency”. Any monogamous mating function is then readily extendable–with preservation of the efficiency–to non‐monogamous systems, so that, for instance, the probability of encounter of one male and one female in the monogamous system corresponds to the probability of “encounter” of one male and *h* females in the corresponding polygynous system (with *h* the mean harem size, Caswell 2001).

For *the minimum* (monogamous) *mating function* (of parameter 1), the number of pairs is set by the less abundant sex, with *f* and *m*, respectively, the number of available females and males:

$$U_{\min,1}\left(m,f\right)=\min\left(m,f\right).$$

This function is mathematically valid (Supporting Information S1) but assumes a 100% efficiency: every individual of the less numerous sex should encounter a mate and reproduce. This is, in general, ecologically unrealistic for populations in the wild where some individuals can be sterile, skip reproduction, or simply not get access to potential mates. For a given number of females *f* and males *m* in the population, the minimum function actually corresponds to the theoretical maximum number of pairs formed, that is, to the maximum possible efficiency in mating (it is 1 across the spectrum of OSR). Two pitfalls of the minimum mating function are that it yields a discontinuous rate of pair formation at the balanced OSR and an efficiency in mating that is independent from the sex ratio, which is also not ecologically realistic (Figure 1A,C).

The *harmonic mean mating function* (of parameter 2) is often considered the most appropriate function in continuous‐time frameworks (Keyfitz 1972; an “ideal” mating function according to Schoen 1981), since it does capture the expected variation in efficiency of the union formation as a function of the OSR: the less relatively abundant a sex is, the more likely an individual of that sex can mate. It has been extended, unchanged, to discrete‐time models (Caswell and Weeks 1986; Caswell 2001):

$$U_{h,2}\left(m,f\right)=\frac{2\mathrm{mf}}{f+m}$$

However, in that framework, it is not mathematically valid because it yields a number of pairs (for all *m* and *f* but for *m = f*) that exceeds the theoretical maximum number allowed by the minimum function. Simply put, it says that the probability to mate of an individual of the limiting sex is higher than 1! This formula has been used in numerous papers and textbooks, some of them influential, concerned with theoretical analyses and applications (e.g., Lindström and Kokko 1998; Ranta and Kaitala 1999; Miller et al. 2011). The harmonic mean function violates a crucial rule identified by Pollak (1986): “*the number of unions involving a sex must not exceed the total number of individuals of that sex*”. This assumption can be satisfied easily by using a lower multiplicator, such as $U_{h,1}\left(m,f\right)=\frac{\mathrm{mf}}{f+m}$ (e.g., Gerber and White 2014; Tsai et al. 2015). However, when there are as many available males as females in the population (balanced sex ratio), this function only forms $\frac{n}{4}$ pairs (with *n* the total number of individuals in the population), and half the males and half the females remain alone. While this captures the fact that the efficiency of union formation is lowest when there are as many males as females, the proportion of unmated individuals is not flexible and too high to be ecologically meaningful (Figure 1A).

The *modified harmonic mean mating function* introduced by Legendre (2004) and applied in empirical studies (e.g. Gerber and White 2014):

$$U_{\mathrm{mh}}\left(m,f\right)=\min\left(f,\frac{2\mathrm{mf}}{f+m}\right)$$

leads to a number of pairs formed that cannot exceed the number of reproductive females (Legendre 2004). In other words, this modified harmonic mean mating function combines the *harmonic mean* and the *minimum mating functions* to account for the problem of the invalid number of pairs when there are more males than females (Figure S1). However, when there are more females than males, the number of pairs exceeds the number of males, and this function is not (and was not designed as such by Legendre 2004) a monogamous mating function that can be extended to polygamous systems.

In this study, we introduce a novel mating function suitable for discrete‐time models, designed to ensure that the number of unions does not exceed the number of reproductive individuals, whether male or female. This new mating function we call “*Minharmonic mating function*” (1) obeys the general rules of continuous‐time mating functions; (2) does not overestimate the number of mating pairs; (3) decreases in mating efficiency (i.e., the probability to mate per individual of the limiting sex) as one approaches the balanced OSR; (4) allows flexibility in how strongly mating efficiency declines upon approach of the balanced OSR via a parameter *e*; and (5) can be extended to other mating systems (polygynous, polyandrous, and promiscuous). Moreover, we formalise the concept of mating efficiency, which enhances the understanding of the behaviour of mating functions and prevents logical fallacies. To illustrate the usefulness of our approach, we model a wild boar (*Sus scrofa*) population in a deterministic context (with no variance amongst individuals allowed except between sexes and amongst size classes), using a two‐sex model including this novel mating function. The wild boar is a polytocous, polygynous (Mauget 1980; Gayet et al. 2021), and size‐dimorphic mammalian species that markedly benefits from current global changes (Touzot et al. 2020, 2023). Managing wild boar populations through hunting has become increasingly challenging due to their significant population growth and expansion across Europe over the last decades (Massei et al. 2015).

## 2 Materials and Methods

### 2.1 A Novel Mating Function for Discrete‐Time Models

#### 2.1.1 Important Properties of Mating Functions

We propose a new mating function that combines the minimum mating function and the harmonic mean mating function (see Box 2 for a description of these functions) and in its monogamous formulation obeys the four required properties set by theoreticians of two‐sex models in a continuous‐time framework to have meaningful interpretation (Fredrickson 1971; Gupta 1972; Yellin and Samuelson 1974):

‐ Monotonicity: the mating function $U\left(m,f\right)$, giving the number of pairs formed in a population with *m* available males and *f* available females, is a non‐decreasing function of *m* and *f*;
‐ Non‐negativity: $U\left(m,f\right)\geq 0$;
‐ No union if one sex is absent;
‐ Homogeneity: if the number of both sexes increases *k* times, the number of unions increases *k* times also.

It also obeys a crucial mandatory property for monogamous models in discrete‐time, given by Pollak (1986):

‐ Mathematical validity: “the number of unions involving a sex must not exceed the total number of individuals of that sex”.

Moreover, it has several desirable properties for mating functions to be ecologically realistic and/or useful for the theoretical development and analysis of two‐sex models:

‐ Symmetry: the mating function is symmetrical with respect to a balanced OSR;
‐ Mating efficiency (i.e., probability to mate) increases with the distance to the balanced sex ratio: the probability to mate for an individual of the limiting sex is smaller (i.e., there is lower mating efficiency) at or around the balanced OSR than away from it;
‐ Continuity and derivability: the mating function and its derivative are continuous in *m* and *f*;
‐ Adaptability to any mating system: it is designed as a monogamous function readily extendable to any mating system (polygyny, as we illustrate with the wild boar case study, polyandry, or promiscuity);
‐ Flexibility: a parameter (*e*) controlling for the efficiency at the OSR and therefore for how strongly mating efficiency declines upon approach of the balanced OSR.

#### 2.1.2 Formulation of the Novel Minharmonic Mating Function in a Monogamous System

A monogamous mating function $U\left(m,f\right)$ yields the number of pairs formed as a function of available males *m* and females *f*. Because of the “homogeneity” property, $U\left(m,f\right)$ can be described equivalently and in a simpler manner as a univariate function $U^{*}\left(f^{*}\right)$ of the relative number of females: $f^{*}=\frac{f}{m+f}$ (i.e., of the OSR). $U^{*}\left(f^{*}\right)$ corresponds to the number of unions per individual in the population:

(1) $$U^{*}\left(f^{*}\right)=\frac{U\left(m,f\right)}{m+f}$$

For example, for the “minimum” function of parameter *p*, commonly used with *p = 1* (Jenouvrier et al. 2010), we have $U_{\min,p}^{*}\left(f^{*}\right)=pmin\left(f^{*},1-f^{*}\right)$. For the “harmonic mean” function of parameter *p*, commonly used with *p = 2* (Caswell 2001), this is $U_{h,p}^{*}\left(f^{*}\right)=pf^{*}\left(1-f^{*}\right)$ (see Box 2 for the full description of these functions).

From a monogamous mating function, we can define the *mating efficiency* as the probability for an individual of the limiting sex “to meet and mate”, corresponding to:

(2) $$E^{*}\left(f^{*}\right)=\frac{U^{*}\left(f^{*}\right)}{\min\left(f^{*},1-f^{*}\right)\;}$$

From Equations (1) and (2), we can write the efficiency as a function of the number of males *m* and females *f*, $E\left(m,f\right)=\frac{U\left(m,f\right)}{\min\left(f,m\right)\;}$.

In particular, we denote *e*, the mating efficiency at the balanced sex ratio: $e=E^{*}\left(0.5\right)=2U^{*}\left(0.5\right)$ (from Equation (2)). The parameter *e* and more generally $E^{*}\left(f^{*}\right)$ are probabilities: they must be positive and smaller than 1.

The minimum mating function of parameter *p = 1* is not ecologically realistic because its efficiency $E_{\min,1}^{*}\left(f^{*}\right)=1$ is independent from $f^{*}$ (see Figure 1C) and too high (all adult individuals reproduce); moreover, the mating function's derivative is not continuous at a balanced sex ratio (see Figure 1A). One can choose a different parameter *p < 1* for the minimum mating function to reduce its efficiency and obtains $E_{\min,p}^{*}\left(f^{*}\right)=p$, which shows that the latter is still independent from the OSR (see the case *p = 0.5* for the minimum function on the Figure 1C). The harmonic mean mating function requires a parameter p≤1 to avoid the violation of the Pollack rule: for *p >* 1 (as for the widely used harmonic function of parameter 2), the probability to mate (i.e., the efficiency) is higher than 1 (see Figure 1C). For p≤1, the harmonic mean mating function is not logically flawed but corresponds to an unrealistically low efficiency (see Figure 1C).

To obey the required properties (see Supporting Information S1 for the full description of each property), our novel mating function is built as a combination of the minimum mating function and a modified harmonic mean mating function. This mixture (and its derivative) must be continuous in each point and the mixing is performed according to the efficiency parameter *e* of the resulting mating function (see Supporting Informations S2 and S3 for the construction of the minharmonic mating function and its effect at the population level). For 0≤e<0.5, the minharmonic mating function is simply the harmonic mean function of parameter *p = 2e* (from $e=E_{h,p}^{*}\left(0.5\right)=0.5*p$), leading to $U_{\text{minh},e}^{*}\left(f^{*}\right)=2ef^{*}\left(1-f^{*}\right)$ (see Figure 1). For 0.5≤e≤1, the minharmonic function corresponds to:

(3) $$U_{\text{minh},e}^{*}\left(f^{*}\right)=\left\{\begin{array}{c} \min\left(f^{*},1-f^{*}\right)\;\text{for}\;f^{*}\leq e-0.5\;\text{and}\;f^{*}\geq 1.5-e\;\\ \frac{f^{*}\left(1-f^{*}\right)-{\left(e-0.5\right)}^{2}}{2\left(1-e\right)}\;\text{for}\;e-0.5\leq f^{*}\leq 1.5-e \end{array}\right.$$

The minharmonic mating function of parameter 0.5≤e≤1, therefore, corresponds to the minimum mating function with *p = 1* for unbalanced OSRs; i.e. all individuals of the limiting sex mate at small $f^{*}$ ($0\leq f^{*}\leq e-0.5$) and symmetrically, at large $f^{*}$ ($0\leq 1-f^{*}\leq e-0.5$). In the extreme case e=1, the minharmonic function is the minimum function of parameter *p = 1* across all $f^{*}$. For balanced OSRs, that is $e-0.5\leq f^{*}\leq 1.5-e$, the minharmonic function has the shape of the harmonic function of parameter $p=\frac{1}{2\left(1-e\right)}$, but is not equal to it (its intercept is not zero), unless in the case e=0.5, where the minharmonic function is the harmonic mean function of parameter *p = 1*. The mating efficiency with the minharmonic function, decreases continuously from $E^{*}\left(0\right)=E^{*}\left(1\right)=1$ to the efficiency at the balanced OSR: $E^{*}\left(0.5\right)=e$ (see Figure 1B and computation of $E^{*}\left(0.5\right)$ in Supporting Information S2).

#### 2.1.3 Formulation of the Minharmonic Mating Function in a Polygynous System

Any correctly built monogamous mating function $U\left(m,f\right)$ can be extended to other mating systems. For a polygynous population with a mean harem size *h*, for example, one gets for the number of harems formed in a population with *m* available males and *f* available females (see Supporting Information S4 for other systems such as polyandry or promiscuity):

(4) $$U^{\mathrm{pg}}\left(m,f,h\right)=U\left(m,\frac{f}{h}\right)=\left(m+\frac{f}{h}\right)U^{*}\left(\frac{f}{\mathrm{hm}+f}\right)$$

For the minharmonic mating function, from Equations (3) and (4) we get:

(5) $$U_{\text{minh},e}^{\mathrm{pg}}\left(m,f,h\right)=\left\{\begin{array}{c} \frac{1}{h}\min\left(f,\mathrm{mh}\right)\text{for}\;\frac{f}{\mathrm{hm}+f}\leq \left(e-0.5\right)\;\text{and}\;\frac{f}{\mathrm{hm}+f}\geq \left(1.5-e\right)\\ \;\frac{\frac{\mathrm{mf}}{\left(\mathrm{hm}+f\right)}-\frac{\left(\mathrm{hm}+f\right)}{h}{\left(e-0.5\right)}^{2}}{2\left(1-e\right)}\;for\;\left(e-0.5\right)\leq \frac{f}{\mathrm{hm}+f}\leq \left(1.5-e\right) \end{array}\;\right.$$

The monogamous mating system is a special case of the polygynous case with h=1 (see the equation (7) from the Supporting Information S2).

Finally, from the polygynous mating function $U_{\text{minh},e}^{\mathrm{pg}}\left(m,f,h\right)$ and *K*, the mean number of offspring produced by a mated female, we can generate the birth functions for females (*Bf*) and for males (*Bm*), which compute the expected number of offspring produced by a female and a male, respectively. These birth functions correspond to the expected number of offspring produced by a mated individual (*K* offspring for a female and *hK* offspring for a male) times the probability to mate ($\frac{h\times U_{\text{minh},e}^{\mathrm{pg}}\left(m,f,h\right)}{f}$ for females and $\frac{U_{\text{minh},e}^{\mathrm{pg}}\left(m,f,h\right)}{m}$ for males):

(6a) $$\mathrm{Bf}\left(m,f,h\right)=K\times h\times \frac{U_{\text{minh},e}^{\mathrm{pg}}\left(m,f,h\right)}{f}$$

(6b) $$\mathrm{Bm}\left(m,f,h\right)=K\times h\times \frac{U_{\text{minh},e}^{\mathrm{pg}}\left(m,f,h\right)}{m}$$

### 2.2 Application to the Case Study of an Intensively Exploited Population of Wild Boar

To illustrate the usefulness of our approach, we built a two‐sex body size‐structured matrix projection model **A** for the polygynous wild boar (see Table 1). Our model was comparable to the one developed by Gamelon et al. (2012), as it includes three body‐size classes for females (small < 30 kg, 30 kg < medium < 50 kg, large > 50 kg), three body‐size classes for males (small < 45 kg, 45 kg < medium < 75 kg, large > 75 kg). It also incorporates sex‐ and size‐specific vital rates (growth, survival, reproduction), which were previously estimated in the population of Châteauvillain‐Arc‐en‐Barrois, France (Gamelon et al. 2012; see Table 2 for the value and biological meaning of all parameters). Over one year, an individual grows towards the size class i directly above its current one, with probability $G_{i}$ (where *i*: *Mf =* medium females, *Mm =* medium males, *Lf =* large females and *Lm =* large males, so $G_{\mathrm{Mf}}$, for instance, is the probability for a small female to be in medium‐size class the following year), or can remain in its size class with probability 1‐*G*_*i*_. Juveniles will, at the end of their first year, be of size medium with probability $G_{\text{juvMf}}$ (for females and $G_{\text{juvMm}}$ for males) or of small size with probability $1-G_{\text{juvMf}}$ (for females and $1-G_{\text{juvMm}}$ for males; see Table 1 for the matrix and Table 2 for the value and biological meaning of parameters).

**TABLE 1:** A two‐sex body size‐structured matrix model **A** for the wild boar population of Châteauvillain, France.

		♀			♂		
		Small	Medium	Large	Small	Medium	Large
♀	Small	(1−*G*_*Mf*_) × *SSF* × (1−*HSf*)	0.5 × 0.5 × *s*0 × ***Bf***_***M,t***_ × (1−*G*_*juvMf*_) × *SSf* × (1−*HSf*)	0.5 × 0.5 × *s*0 × ***Bf***_***L,t***_ × (1−*G*_*juvMf*_) × *SSf* × (1−*HSf*)	0	0.5 × 0.5 × *s*0 × ***Bm***_***t***_ × (1−*G*_*juvMf*_) × *SSf* × (1−*HSf*)	0.5 × 0.5 × *s*0 × ***Bm***_***t***_ × (1−*G*_*juvMf*_) × *SSf* × (1−*HSf*)
	Medium	*G*_*Mf*_ × *SMf* × (1−*Hf*)	((1−*G*_*Lf*_) + 0.5 × 0.5 × *s*0 × ***Bf***_***M,t***_ × *G*_*juvMf*_) × *SMf* × (1−*Hf*)	0.5 × 0.5 × *s*0 × ***Bf***_***L,t***_ × *G*_*juvMf*_ × *SMf* × (1−*Hf*)	0	0.5 × 0.5 × *s*0 × ***Bm***_***t***_ × *G*_*juvMf*_ × *SMf* × (1−*Hf*)	0.5 × 0.5 × *s*0 × ***Bm***_***t***_ × *G*_*juvMf*_ × *SMf* × (1−*Hf*)
	Large	0	*G*_*Lf*_ × *SLf* × (1−*Hf*)	*SLf* × (1−*Hf*)	0	0	0
♂	Small	0	0.5 × 0.5 × *s*0 × ***Bf***_***M,t***_ × (1−*G*_*juvMm*_) × *SSm* × (1−*HSm*)	0.5 × 0.5 × *s*0 × ***Bf***_***L,t***_ × (1−*G*_*juvMm*_) × *SSm* × (1−*HSm*)	(1−*G*_*Mm*_) × *SSm* × (1−*HSm*)	0.5 × 0.5 × *s*0 × ***Bm***_***t***_ × (1−*G*_*juvMm*_) × *SSm* × (1−*HSm*)	0.5 × 0.5 × *s*0 × ***Bm***_***t***_ × (1−*G*_*juvMm*_) × *SSm* × (1−*HSm*)
	Medium	0	0.5 × 0.5 × *s*0 × ***Bf***_***M,t***_ × *G*_*juvMm*_ × *SMm* × (1−*HMm*)	0.5 × 0.5 × *s*0 × ***Bf***_***L,t***_ × *G*_*juvMm*_ × *SMm* × (1−*HMm*)	*G*_*Mm*_ × *SMm* × (1−*HMm*)	((1−*G*_*Lm*_) + 0.5 × 0.5 × *s*0 × ***Bm***_***t***_ × *G*_*juvMm*_) × *SMm* × (1−*HMm*)	0.5 × 0.5 × *s*0 × ***Bm***_***t***_ × *G*_*juvMm*_ × *SMm* × (1−*HMm*)
	Large	0	0	0	0	*G*_*Lm*_ × *SLm* × (1−*HLm*)	*SLm* × (1−*HLm*)

*Note:* Three size classes are considered: small (< 30 kg for females, < 45 kg for males), medium (between 30 and 50 kg for females; between 45 and 75 for males), and large (> 50 kg for females; > 75 kg for males). Birth functions for a given year *t* are ${\mathrm{Bf}}_{M,t}$ for medium females, ${\mathrm{Bf}}_{L,t}$ for large females, and ${\mathrm{Bm}}_{t}$ for males. Birth functions (in bold) are fully described in the main text. See Table 2 and Gamelon et al. (2012) for the value and biological meaning of each parameter.

**TABLE 2:** Parameters, their biological meanings, and values (in rows) estimated by Gamelon et al. (2012) and used in the matrix population model **A**.

Parameter	Biological meaning	Value estimated
SSf	Natural survival of small females	0.978
SMf	Natural survival of medium females	0.855
SLf	Natural survival of large females	0.859
HSf	Proportion of small females killed by hunting	0.449
SSm	Natural survival of small males	0.962
SMm	Natural survival of medium males	0.777
SLm	Natural survival of large males	0.904
HSm	Proportion of small males killed by hunting	0.511
HMm	Proportion of medium males killed by hunting	0.541
HLm	Proportion of large males killed by hunting	0.789
G_Mf_	Probability for a small female to grow in medium size class	0.879
G_Lf_	Probability for a medium female to grow in large size class	0.569
G_Mm_	Probability for a small male to grow in medium size class	0.747
G_Lm_	Probability for a medium male to grow in large size class	0.678
G_juvMf_	Probability for a juvenile female to grow in medium size class	0.4
G_juvMm_	Probability for a juvenile male to grow in medium size class	0.4
s0	Postnatal survival	0.75
*K*_*M*_	Mean number of juveniles produced by a medium female	5
*K*_*L*_	Mean number of juveniles produced by a large female	6

During the non‐hunting period (from March to September), each individual has a natural survival probability depending on its size class (see Table 2). During the hunting period (from October to February), the proportion of individuals killed in each size class for each sex is denoted as HSf for small females, and Hf for other females (medium and large females are considered to be killed in the same proportion), and HSm,HMm,HLm for small, medium and large males, respectively. The probability not to be killed by hunters corresponds to 1−H, with H the proportion of individuals killed (see Supporting Information S5 for the full description of the matrix). Small‐sized individuals are not sexually mature. Medium and large individuals can reproduce, and medium females produce *K*_*M*_ = 5 offspring per litter, while large females produce on average *K*_*L*_ = 6 offspring per litter (Gamelon et al. 2012). The young produced have a postnatal survival of *s0* (see Table 2). In contrast to Gamelon et al. (2012) that included both alive and dead individuals, our model here only includes alive individuals, and more importantly, includes the novel minharmonic mating function.

We denote *f* and *m* as the number of sexually mature females and males in total, respectively (i.e., *m* = *m*_*M*_ + *m*_*L*_ with *m*_*M*_ the number of medium males and *m*_*L*_ the number of large males; and *f* = *f*_*M*_ + *f*_*L*_ with *f*_*M*_ the number of medium females and *f*_*L*_ the number of large females). Each breeding male mates with *h* females. In our mating function, we assume that males in unions with *h* females are not available for other females. The minharmonic function controls for the number of unions, leading to the following birth functions for body‐size classes of females (${\mathrm{Bf}}_{M}$ for medium females and ${\mathrm{Bf}}_{L}$ for large females) and for males (Bm) for a harem size *h* (see Supporting Information S6 for the full description of the size‐structured birth functions):

$${\mathrm{Bf}}_{M}=K_{M}\times h\times \frac{U_{\text{minh},e}^{\mathrm{pg}}\left(m,f,h\right)}{f}$$

$${\mathrm{Bf}}_{L}=K_{L}\times h\times \frac{U_{\text{minh},e}^{\mathrm{pg}}\left(m,f,h\right)}{f}$$

$$\mathrm{Bm}=\frac{f_{M}K_{M}+f_{L}K_{L}}{f}\times h\times \frac{U_{\text{minh},e}^{\mathrm{pg}}\left(m,f,h\right)}{m}$$

In the two‐sex projection model (Table 1), we included the sex ratio at birth (balanced, see Servanty et al. 2007), and, since an offspring is produced by both a male and a female, we used half of the birth rates as fertility rates (Table 1), so that offspring are only counted once.

We projected the wild boar population size over a T = 20 year‐period using the following equation: $N_{t+1}=AN_{t}$, where $N_{t}$ is the population vector describing the number of individuals in each size class *z* (S: small, M: medium and L: large) for each sex at time *t* and **A** is the population matrix of Table 1. Birth functions depend on the number of available breeding individuals at time *t*, so we estimated ${\mathrm{Bf}}_{M,t}$, ${\mathrm{Bf}}_{L,t}$, and ${\mathrm{Bm}}_{t}$ for a given year *t*. We initiated the simulations with the following population vector: *f*_*S*_ = *m*_*S*_ = 31, *f*_*M*_ = *m*_*M*_ = 92 and *f*_*L*_ = *m*_*L*_ = 154, which corresponds to the average number of females observed post‐hunt and before births in the field, in each size class in this population (see Gamelon et al. 2021). We approximated the asymptotic population growth rate λ, by $\lambda \approx \lambda \left(T\right)=\frac{N_{\mathrm{tot}}\left(T+1\right)}{N_{\mathrm{tot}}\left(T\right)}$, with $N_{\mathrm{tot}}=\sum_{z}\left(f_{z}+m_{z}\right)$; as after T = 20 time‐steps, in all cases, the transient dynamics had dissipated and the two‐sex size‐structured relative abundances corresponded to equilibrium values.

**FIGURE 2:**
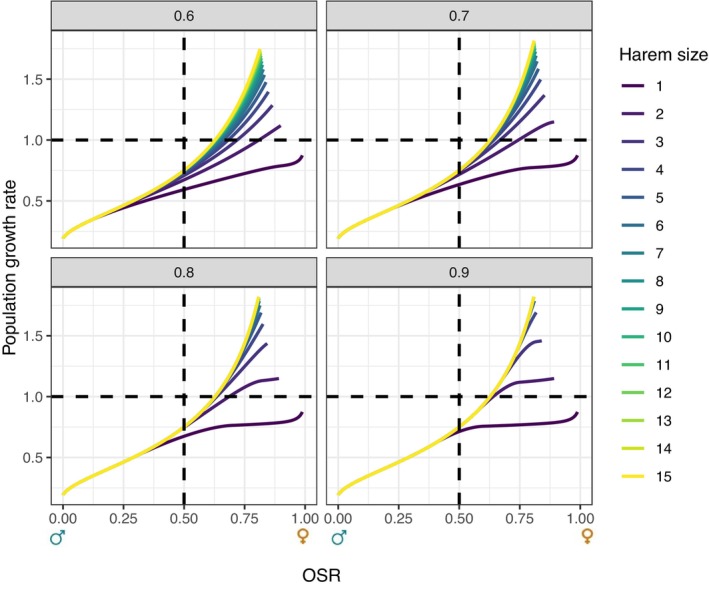
Population growth rate in relation to the operational sex ratio (OSR) in response to various hunting scenarios, for the wild boar population modelled with the minharmonic mating function with different harem sizes *h* (different colours) and efficiency *e* (various plots).

At this stable state, we calculated the asymptotic OSR as $\;\frac{f\left(T\right)}{\left(m\left(T\right)+f\left(T\right)\right)}$. To explore the influence of a change in the OSR on the population growth rate, we incorporated different hunting scenarios targeting varying proportions of the reproductive females. To do so, we kept all other vital rates constant and had the proportion of sexually mature females killed by hunters Hf, vary from 0 (i.e., leading to a sex ratio in favour of females as only males are harvested) to 1 (i.e., leading to a sex ratio in favour of males as only breeding females are shot by hunters). For a given mating efficiency at the balanced OSR, *e* and harem size *h*, a given hunting scenario yields therefore a unique OSR and a unique λ at the equilibrium. We projected the population for different values of *e* (from 0.6, a weak efficiency in mating, to 0.9, a high efficiency in mating), and over different harem sizes (from *h* = 1 to *h* = 15) and plotted the results in Figure 2.

Additionally, to compare the application of our minharmonic mating function to alternative mating functions, we projected the wild boar population and approximated the population growth rate, for a monogamous mating system (i.e., *h = 1*), across the three mating functions we have focused on (i.e., minharmonic, harmonic mean and minimum), standardised to the same efficiency in mating at the balanced sex ratio: $e=E^{*}\left(0.5\right)=2\times U^{*}\left(0.5\right)=0.8$. For the minimum mating function, this corresponds to a parameter *p = 0.8* as $E_{\min,p}^{*}\left(f^{*}\right)=p$. For the harmonic mean mating function, this corresponds to a parameter *p =* 1.6 as $E_{h,p}^{*}\left(f^{*}\right)=pmax\left(f^{*},1-f^{*}\right)$ and therefore $E_{h,p}^{*}\left(0.5\right)=0.5\times p$. Note that the harmonic function with *p* = 1.6 corresponds to a mating function that violates mandatory property 8 (with mating probability higher than 1; see Supporting Information S1). We did the same thing for e=0.9.

All analyses were performed with R (v.4.1.3, R Core Team 2021).

## 3 Results of the Application to the Wild Boar Case Study

As expected, hunting scenarios impacted the OSR through a change in female wild boar survival, and, together, vital rates, and OSR had major effects on the population growth rate. For a given OSR, the population growth rate increased with both the efficiency and the harem size (see Figure 2 and see Supporting Information S7 for the relation between Hf, the OSR and the population growth rate). For instance, for a population with an OSR of 0.75 (i.e., 3 mature females for 1 mature male, that corresponds to a hunting strategy with a low number of females killed leading to a high number of reproductive females) and a low efficiency in mating (i.e., *e* = 0.6, top left quadrant of Figure 2), the population growth rate varied from 0.72 for a monogamous mating system to 1.40 for a polygynous mating system with a harem size of 15 (i.e., 15 females for 1 male). For the same OSR, but with a high efficiency in mating (i.e., *e* = 0.9, bottom right quadrant of Figure 2), the population growth rate ranged from 0.77 for a monogamous mating system to 1.42 for a polygynous mating system with a harem size of 5 or more (i.e., 5 or more females for 1 male; see Figure 2). In this case, where OSR is strongly female‐biased ($f^{*}=0.75$), and mating efficiency is high (*e = 0.9*), the population growth rate is constant for any h>5. This is because the harem size is then sufficiently large for the $\frac{f}{f+\mathrm{mh}}$ sex ratio to correspond to the minimum mating function (of parameter 1) component of the minharmonic, where all females reproduce (we are in the case $f\leq \left(e-0.5\right)\left(f+\mathrm{mh}\right)$ of Equation (5)).

In contrast, for an OSR biased in favour of males (i.e., high hunting mortality for females and OSR lower than 0.5), the population growth rate was lower than 1 (in all cases, see Figure 2), leading to a declining population size. For example, for a population with a sex ratio of 0.25, and with a low efficiency in mating (i.e., *e* = 0.6), the population growth rate varied from 0.44 for a monogamous mating system, to 0.46 for a polygynous mating system with a harem size of 3 (i.e., 3 females for 1 male). For the same OSR, but with a high efficiency in mating (i.e., *e* = 0.9), the population growth rate did not vary amongst mating systems or harem sizes (λ = 0.46; Figure 2). Even though, here again, all adult females reproduced, λ was small due to a high hunting mortality of females, resulting in a reduced number of reproductive females.

For extreme sex ratios, for instance, an OSR of 0.99 (i.e., extremely biased in favour of females), the asymptotic population growth rate is estimated at 0.87 for a monogamous mating system, corresponding to a declining population whatever the value of *e*. At the opposite, for a sex ratio of 0.01 (i.e., extremely biased in favour of males), the asymptotic population growth rate is estimated at 0.20 for a monogamous mating system, whatever the value of the efficiency in mating, leading the population to extinction very quickly.

Overall, sex‐specific survival rates that can be modified by more or less selective hunting pressure strongly impact the number of reproductive individuals, and therefore the OSR. This OSR has a strong impact on the population growth rate, leading to population size increases when OSR is in favour of females. However, the mating efficiency does not change the general pattern of the variation in the population growth rate to the OSR, but it modifies the harem size needed to reach the highest population growth rate (Figure 2).

**FIGURE 3:**
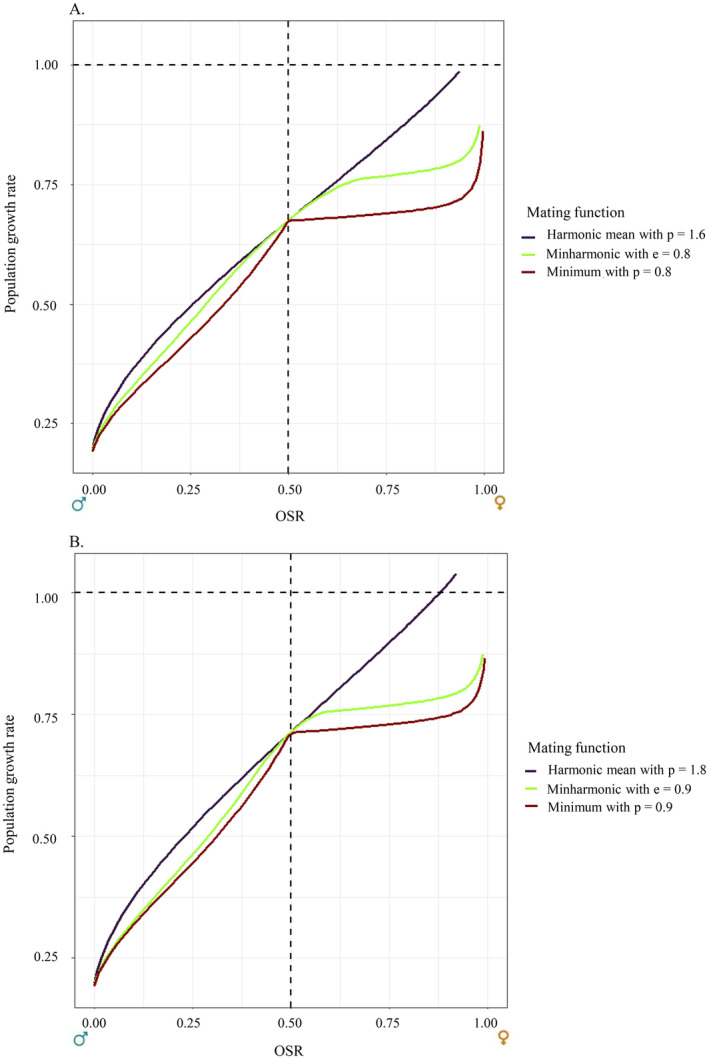
Comparison of the effects of the minimum, harmonic mean, and minharmonic mating functions, standardised for $E^{*}\left(f^{*}=0.5\right)=0.8$ (A) and $E^{*}\left(f^{*}=0.5\right)=0.9$ (B), on wild boar population growth rate for a monogamous mating system (i.e., *h = 1*).

The response of the population growth rate to various survival scenarios leading to various OSR shows that the three mating functions we have studied (i.e., the minimum function, the harmonic mean and the minharmonic) have very different behaviours and influence population dynamics differently (see Figure 3). This is true even for OSRs that are close to $f^{*}=0.5$, which should allow distinguishing a best fit between these functions when operating statistical model selection from data.

## 4 Discussion

Modelling a two‐sex population using the novel minharmonic mating function allows accounting for (1) sex‐specific vital rates, (2) the number of available breeding individuals in the population, (3) various mating systems, and (4) flexibility in mating efficiency and how this efficiency varies with the OSR. Contrary to commonly used mating functions, the flexible minharmonic mating function obeys all properties, mandatory and desirable, for a mating function to be mathematically valid and ecologically meaningful. As expected and illustrated with the wild boar case study, the OSR largely influences population growth rate, highlighting the importance of appropriately modelling unions producing offspring.

Social monogamy is displayed by about 90% of bird species and approximately 3% of mammalian species (Lack 1968). In these species, one reproductive male per reproductive female is required to produce offspring. Incorporating the number of unions formed by males and females without overestimating that value is critical to obtain accurate estimates of the Malthusian fitness from two‐sex population models. The mating function we introduce here rights the wrongs of numerous past studies by using the “harmonic mean” mating function (of parameter 2) in a discrete‐time framework. This novel mating function, like the “harmonic mean” one, produces a union formation that increases in mating probability for the less numerous sex as one moves away from the balanced OSR, but avoids the major pitfall of yielding more pairs than the number of available males and females. The minharmonic function has one parameter (i.e., the mating efficiency at the balanced OSR, *e*) that mixes its two underlying components: the minimum mating function for extreme OSRs (either large or small) and a modified harmonic mean function for balanced OSRs. Since the harmonic component has a non‐zero intercept, the behaviour of the minharmonic mating function differs markedly from both the minimum and the harmonic functions (even when scaled by a parameter to have equal *e*, see Figure 3), allowing for statistical assessment of the function that best fits the data. The minharmonic mating function is readily extendable to other mating systems, as we showed with the case study of a polygynous wild boar population intensively hunted (see Supporting Information S4 for polyandry and promiscuity).

As for monogamous species, in the case of polygyny characterised by a harem size *h* greater than 1, it is crucial to properly incorporate the correct number of unions. We showed that the population growth rate is not solely dependent on *h*, but also on the value of *e*, representing mating efficiency. When *e* is low, increasing the harem size leads to a sharp increase in population growth rate. However, when mating efficiency is high, even a small harem size is sufficient to achieve high population growth rates (Figure 2). It is noteworthy that while *e* affects population dynamics, it has little effect on the general pattern of variation in the population growth rate to the OSR, compared to harem size and vital rates themselves. Strikingly, two‐sex models accounting for polygynous mating systems may be an interesting tool to estimate the population growth rate across a wide range of harem sizes, even in the absence of prior biological knowledge about *h*, as long as there is some understanding of the OSR of the population.

The OSR may vary as a result of changes in management or conservation strategies. In exploited populations subject to sex‐ or size‐selective harvesting, the OSR can be biased in favour of one sex, directly influencing the number of unions between reproductive individuals. In this study of a wild boar population, we showed that when females were less intensively hunted than males, a biased OSR in favour of females led to an increase in population size, while the opposite was observed for a biased OSR in favour of males (see Figure 2). In the case of endangered populations, considering the OSR and the mating system using a relevant mating function can also help provide valuable recommendations for managers or conservationists. For example, in the polygynous saiga antelope (*Saiga tatarica tatarica*), classified as “critically endangered” by the IUCN due to intensive poaching of males for their horns, the OSR was strongly skewed in favour of females. With too few adult males, many females were unable to find a mate, resulting in a sharp decline in population size (Milner‐Gulland et al. 2003). Here, a two‐sex population model including the minharmonic mating function for polygynous mating system would help develop effective conservation measures. Our minharmonic mating function is a relevant tool for considering mating system and the number of reproductive individuals in two‐sex models, making it widely applicable to monogamous, polygynous, polyandrous, or promiscuous mating systems (see Supporting Information S4). Moreover, it includes mating efficiency explicitly as a parameter (*e*), contrary to most standard functions for which (implicit) efficiency is rarely computed. The commonly used “minimum” mating function (of parameter 1) corresponds in fact to a very high efficiency (i.e., 100% of the adult individuals that can reproduce), while the “harmonic mean” of parameter 1 corresponds to a rather weak efficiency (i.e., only 50% of the adult individuals reproduce in the population at the balanced OSR), while the classic “harmonic mean” (of parameter 2) corresponds to probabilities to mate higher than 1 (see Figure 1A,C). The minharmonic mating function allows incorporating realistic levels of efficiency and is versatile by fixing this efficiency to a parameter *e* (see Figure 1B,D). Depending on the species or the population, mating efficiency can vary greatly, which affects strongly the population growth rates (Figure 2).

A flexible and straightforward approach to account for the influence of OSR and mating system is crucial to realistically model population dynamics in the wild, particularly in the current context of climate change. For instance, a shift from one mating system to another can be observed in response to changing environmental conditions (Byers and Kitchen 1988; Lane, Forrest, and Willis 2011). In the Bering Sea, the occurrence of El Niño events has deleterious consequences on seabird species like kittiwakes (*Rissa tridactyla*), the main food resource for the red fox (*Vulpes vulpes*). As a result, the diet of the red fox shifts to alternative food resources such as parakeet auklets (*Aethia psittacula*) and rodents, inducing a change in red fox habitat use, which in turn affect the probability to find mates, ultimately causing a shift from polygyny to monogamy (Zabel and Taggart 1989). Evidence for environmentally induced intraspecific variation in mating tactics (e.g., lek or assortative mating) is also accumulating (Jackson 1978). In western Montana, USA, an especially harsh winter causing high mortality in adult male pronghorns (*Antilocapra americana*) led to a change in population age structure that caused a shift in mating tactic from territorial males to non‐territorial ones (Byers and Kitchen 1988). All these environmentally induced shifts in mating systems or tactics can influence population dynamics. For instance, the change in red fox mating system in our first example led to a decline in fox productivity through an increase in the proportion of non‐breeding females and a decrease in reproductive success (Zabel and Taggart 1989). For wild boar, the mating system is also expected to shift from polygyny to promiscuity in response to changes in harvesting regimes. Indeed, the marked decrease in the number of large reproductive males in heavily hunted populations leads to multi‐paternity (e.g., with on average 2.28 fathers within a litter, Gayet et al. 2016; Gamelon et al. 2018). The occurrence of multi‐paternity indicates a shift towards promiscuity under high hunting pressure (Gayet et al. 2021).

## 5 Conclusions

For populations with sex‐specific vital rates and/or where the number of males influences female vital rates, we argue that two‐sex models integrating mating systems should be considered in demographic studies. In the current context of global change where the mating system, the sex ratio, the harem size, or the efficiency in mating can change due to habitat fragmentation (Martin and Martin 2007), exploitation, or a shift in spatial resources' distribution (Zabel and Taggart 1989), the use of accurate, robust and flexible mating functions, such as the minharmonic function proposed here, could improve estimates of the population growth rates and help provide relevant management or conservation strategies. Noticeably, our minharmonic mating function can be straightforwardly applied to various mating systems, including monogamous, polyandrous, or any polygynous species. In addition, while we modelled a mating pattern at random, our approach can be fine‐tuned to account for assortative mating by allowing individuals of similar size or age to mate preferentially together. Moreover, the flexibility of this novel mating function can be useful for any species with sexual reproduction and an OSR that can vary (e.g., vertebrates, insects (e.g., Miller and Inouye 2011), or dioecious plants (e.g., Timerman and Barrett 2020)). Finally, while we modelled the influence of the number of males available on female reproduction, the number of males may also influence female survival in some taxa. For instance, harassment of females by males may reduce adult female survival (Le Galliard et al. 2005; Réale, Boussès, and Chapuis 1996). This can easily be included in the two‐sex model using the same approach as for reproduction.

## Author Contributions

J.C., M.G., and J.‐M.G. designed the study and developed the conceptual ideas with contributions of A.C. and E.B., C.C. developed the theoretical models. J.C. applied the theoretical models to empirical wild boar population data and harvest simulations with the significant contributions of M.G. and C.C., J.C. created visual presentations of the results and wrote the original manuscript. E.B., E.R., and A.C. provided funding. All co‐authors reviewed and contributed substantially to earlier versions of this manuscript.

## Conflicts of Interest

We declare we have no competing interests.

### Peer Review

The peer review history for this article is available at https://www.webofscience.com/api/gateway/wos/peer‐review/10.1111/ele.70013.

## Supporting information


Appendix S1.

## Data Availability

The R codes that support the findings of this study are openly available in Zenodo at https://doi.org/10.5281/zenodo.13938758.
