# Supplementary material for: A Robust and Versatile Mating Function for Two‐Sex Population Projection Models Fitting all Types of Mating Systems
Source: Ecol Lett. 2024 Dec 2;27(11):e70013. doi: 10.1111/ele.70013 (PMC11612543; doi:10.1111/ele.70013)
Supplement: Supplementary file 1 — Appendix S1. [file ELE-27-0-s001.docx]

Ecology Letters

Letter

A robust and versatile mating function for two-sex population projection models fitting all types of mating systems

Jessica Cachelou^1,2,3^, Christophe Coste^4^, Jean-Michel Gaillard^1^, Agathe Chassagneux^2^, Emmanuelle Richard^2^, Eric Baubet^3^, Marlène Gamelon^1^.

1. Laboratoire de Biométrie et Biologie Evolutive, UMR 5558, CNRS, Université Lyon 1, Villeurbanne, France.

2. Fondation François Sommer, Pôle Nature, 3e arrondissement de Paris, France.

3. Office Français de la Biodiversité, DRAS-Service conservation et gestion des espèces à enjeux, Montfort, 01330 Birieux, France.

4. Department of Biosciences, Swansea University, Swansea, United Kingdom.

Corresponding author: Jessica Cachelou, [jessica.cachelou@gmail.com](mailto:jessica.cachelou@gmail.com)

**Supplement 1.** Mandatory properties for continuous framework and desirable properties for discrete framework

We denote $U(m,f)$ a general monogamous mating function, where for *m* and *f* available males and females, respectively, there are $U\left( m,f \right)$ pairs formed.

Early, four properties have been identified for a monogamous union formation in a continuous time framework to have a “meaningful interpretation” (Frederickson 1971; Das Gupta 1972, Yellin and Samuelson 1974):

**Mandatory property 1:** non-negativity: the function $U(m,f)$ is well defined and $U(m,f)\geq0$ for all $m\geq0$ and $f\geq0$, with *m* the number of available males and *f* the number of available females.

**Mandatory property 2:** $U\left( 0,f \right)=U\left( m,0 \right)=0$; if one sex is absent from the population, there is no union.

**Mandatory property 3:** homogeneity: $U\left( km,kf \right)=kU\left( m,f \right)$; if the number of males and females is increased *k* times, the number of unions is increased *k* times also (mathematically, $U$ is a homogeneous function of degree one).

**Mandatory property 4:** monotonicity: if $a\geq0$ and $b\geq0$, $U(m+a, f+b)\geq U(m,f)$, that is, $U$ is a non-decreasing function of *m* and *f*.

Hölder means were highlighted as obeying these criteria by Hadeler (1989) and among those,

- the “minimum” mating function (of parameter 1), $U_{min,1}\left( m,f \right)=min(m,f)$
  - the “minimum” function of parameter *p* being $U_{min,p}\left( m,f \right)=p.min(m,f)$
- and the “harmonic mean” mating function (of parameter 2), $U_{h,2}\left( m,f \right)=\frac{2mf}{m+f}$
  - the “harmonic mean” function of parameter *p* being $U_{h,p}\left( m,f \right)=\frac{pmf}{m+f}$ ,

drew strong interest (both obey obviously the mandatory properties 1-4), and other Hölder mean mating function, such as the geometric mean have also been used (Miller et al. 2011).

It is possible to simplify the expression of bivariate mating function $U\left( m,f \right)$ by noting $n=m+f$, the total population size and $f^{*}=\frac{f}{n}$ and $m^{*}=\frac{m}{n}$, the proportions of females and males in the population (sometimes used as a measure of the operational sex ratio (OSR), as we do in the main text, while others use $\frac{f}{m}=\frac{f^{*}}{m^{*}}$). It yields a univariate function:

$$U^{*}\left( f^{*} \right)=\frac{U\left( m,f \right)}{m+f} \left( 1 \right)$$

which corresponds to the number of unions per individual in the population as a function of the proportion of females (or sex ratio) $f^{*}$. It is the homogeneity (of degree 1, mandatory property 3, above) that allows having $U^{*}=\frac{U}{n}$, independent from *n* and function of $f^{*}$only. $U^{*}$ is defined on $\left[ 0,1 \right]$.

For the “minimum” function, we get $U_{min,1}^{*}\left( f^{*} \right)=min(f^{*}, 1-f^{*})$.

For the “harmonic mean” function, we have $U_{h,2}^{*}\left( f^{*} \right)=2f^{*}( 1-f^{*})$.

Mandatory property 1 translates to $U^{*}\left( f^{*} \right)>0$ for $0\leq f^{*}\leq1$.

Mandatory property 2 becomes $U^{*}\left( 0 \right)=U^{*}\left( 1 \right)=0$.

We plot $U^{*}\left( f^{*} \right)$ for the “harmonic mean” function (in red) of parameter 2 (solid line) and 1 (dotted line), as well as for the “minimum” function (in blue) of parameter 1 (solid line) and 0.5 (star line) on Figure S1.


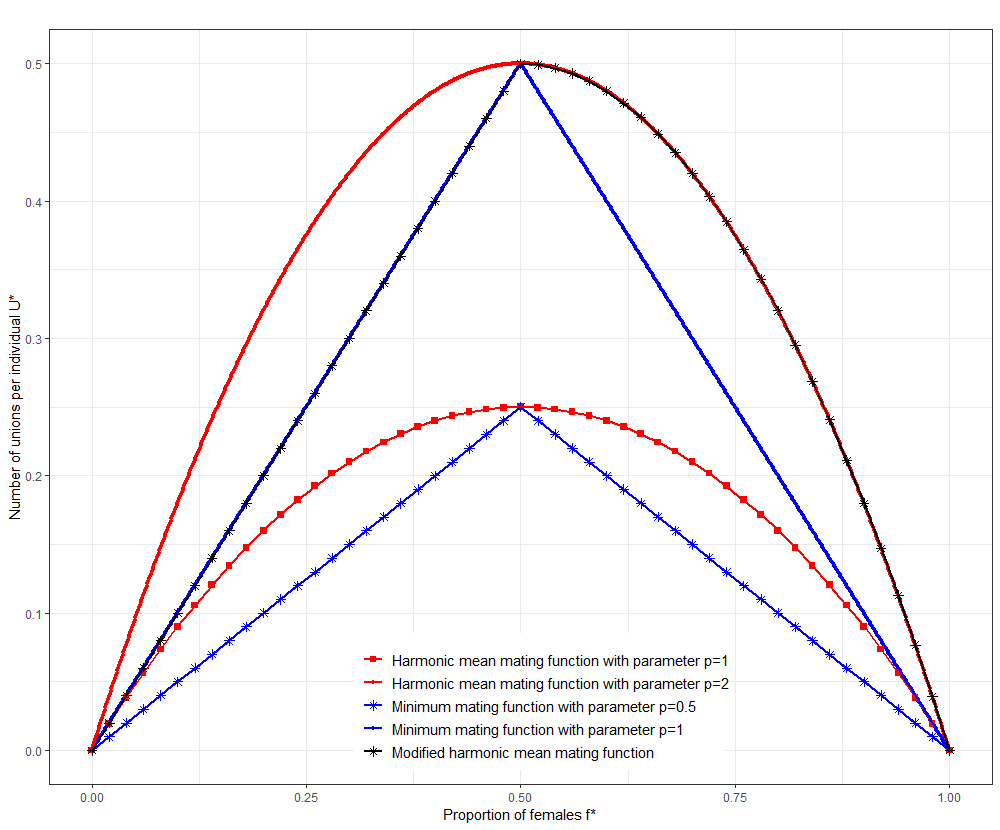


Figure S1. $U^{*}\left( f^{*} \right),$number of pairs (unions) formed per individual as a function of the OSR (proportion of females, $f^{*})$ for various monogamous mating functions.

The shape of these two curves (i.e. minimum and harmonic mean) highlights another set of properties, not mandatory, but desirable:

**Desirable property 5:** symmetry: in the absence of asymmetry in the union formation process, we expect the mating function itself to be symmetrical, i.e. $U\left( m,f \right)=U(f,m)$, which translates into $U^{*}\left( f^{*} \right)=U^{*}\left( {1-f}^{*} \right)$. In other words, $U^{*}$ is symmetrical with regards to $f^{*}=\frac{1}{2}$, the balanced operational sex ratio.

**Desirable property 6a:** continuity: $U(m,f)$ is continuous in *m* and *f*; a property pointed out by Pollack (1990) as mandatory for the population to reach the equilibrium. This property is also related to the ecology: in the absence of discontinuities (e.g. as a specific “regime” would occur for *m* dropping under a certain threshold for instance), $U(m,f)$ has to be continuous too. This translates as $U^{*}\left( f^{*} \right)$ being continuous in $f^{*}$. In fact, in the absence of ecological reasons justifying the contrary, we expect the speed at which the number of unions varies as a function of the proportion of females $f^{*}$ to be continuous too, which yields:

**Desirable property 6b:** derivability and continuous derivatives: the derivative of $U^{*}\left( f^{*} \right)$ exists and is continuous.

We note directly here that $U_{min,p}^{*}(f^{*})$ does not obey the property 6b (for all values of parameter *p*). As the proportion of females overpasses $\frac{1}{2}$, the mating function encounters a shift: $\frac{dU^{*}}{df^{*}}=p$ for $0\leq f^{*}\leq\frac{1}{2}$ and $\frac{dU^{*}}{df^{*}}=-p$ for $\frac{1}{2}\leq f^{*}\leq1$ (see Figure S1). This can be problematic for theoretical development, for example for mathematical integrations.

The “minimum” mating function is also problematic as the probability to mate for an individual of the limiting sex is independent from the sex ratio. Indeed, when modelling potential unions, a classic starting point is to consider them as strongly related to the total number of possible unions between *m* males and *f* females which is $mf=n^{2}f^{*}\left( 1-f^{*} \right)$. We find this quantity, for instance, in the numerator of the “harmonic mean” mating function (one of the reasons why it has been deemed the “best” marriage function (Schoen 1981; Caswell 2001)). In terms of $U^{*}\left( f^{*} \right)$, this implies that it contains an $f^{*}\left( 1-f^{*} \right)$ element as in $U_{h,p}^{*}\left( f^{*} \right)$.

From this consideration, we define the efficiency of a (monogamous) mating function as the probability to mate per individual of the limiting sex (equation (2) in main text):

$$E^{*}\left( f^{*} \right)=\frac{U^{*}\left( f^{*} \right)}{\min\left( f^{*},1-f^{*} \right)} (2)$$

For the minimum function, this leads to $E_{min,p}^{*}\left( f^{*} \right)=p$: the efficiency is independent from the sex ratio, whilst for the harmonic mean function we have $E_{h,p}^{*}\left( f^{*} \right)=pmax(f^{*}, 1-f^{*})$ which indeed decreases when one approaches the balanced sex ratio $( f^{*}=0.5)$ (see Figure 1.C in the main text). This leads naturally to the following desirable property:

**Desirable property 7:** the efficiency $E^{*}\left( f^{*} \right)$ decreases as the proportion of the limiting sex –

that is, $\min\left( f^{*},1-f^{*} \right)$ – increases, leading to a minimal efficiency at a balanced sex ratio (for all $f^{*}, E^{*}\left( f^{*} \right)\geq E^{*}\left( 0.5 \right)=e$).

As it validates all the properties 1-7, it seems that the “harmonic mean” function is the ideal candidate for a mating function for a discrete-time two-sex models. It has been advertised as such by Caswell (2001) and used in many theoretical analyses and applications (e.g. Caswell and Weeks 1986, Lindstrom and Kokko 1998, Ranta and Kaitala 1999, Miller et al. 2011). In reality, this function is far from ideal as these books and papers have overlooked a simple but obvious rule. As they directly transferred the mandatory rules 1-4 from early papers on continuous-time two-sex models (e.g. Frederickson 1971) for studies of discrete-time two-sex models (as is done in Caswell 2001), they forgot a simple and self-explanatory but crucial rule for discrete-time models that the harmonic mean function does not fulfil.

**Mandatory property 8:** There cannot be (in a monogamous union mating function) more unions (pairs) than there are available males or females in the population.

That is for all *m*, *f*, $U(m,f)\leq min(f,m)$. This translates into, for all $f^{*}$, as $U^{*}\left( f^{*} \right)\leq\min\left( f^{*},1-f^{*} \right)$. We note that this corresponds to $\min\left( f^{*},1-f^{*} \right)=U_{min,1}^{*}\left( f^{*} \right)$: the “minimum” function of parameter 1 is the maximum number of unions that any monogamous mating function can produce, in order to be legitimate. Clearly, from Figure S1, this is violated by the “harmonic mean” function of parameter 2 (the red plain curve is above the blue plain one for most of the OSRs). This can be easier seen by translating this principle in terms of mating efficiency: for all $f^{*},$ $E^{*}\left( f^{*} \right)\leq1$, which simply says that a probability (the mating efficiency is the probability to mate) cannot be higher than 1. From $E_{h,p}^{*}\left( f^{*} \right)=pmax(f^{*}, 1-f^{*})$, we can see that for any *p>1*, the “harmonic mean” function of parameter *p* produces probabilities higher than 1, leading to a logical contradiction, and therefore violates mandatory property 8. The mandatory property 8 has actually been stated early by Pollack (1986, 1987, 1990) who wrote: “*The number of unions involving females (males) […] must not exceed the total number of females (males) […] in the population*” (Pollack 1986).

When property 8 is not met, the monogamous union formation is actually a polygamous union formation at all $f^{*}$ where $U^{*}\left( f^{*} \right)>U_{min,1}^{*}\left( f^{*} \right)$. For the “harmonic mean” mating union, Figure S1 clearly shows that $U_{h,2}^{*}\left( f^{*} \right)>U_{min,1}^{*}\left( f^{*} \right)$ at all values of $f^{*}$ but for $f^{*}=\frac{1}{2}$. There is therefore unwanted polyandry on the left-hand side ($f^{*}<\frac{1}{2}$) and unwanted polygyny on the right-hand side ($f^{*}>\frac{1}{2}$). Discrete-time models using this mating function in a monogamous framework are therefore erroneous and their outputs must be reconsidered entirely. In essence, in all these papers, there are more monogamous unions that they are available males or females before the union formation. Of course, if a rule already contains (hidden and uncontrolled) polygamy, it cannot be extended for polygamous unions. To the contrary, if a monogamous mating function is sound and obeys all the mandatory properties, then it can be extended, simply, to non-monogamous mating systems (see Supplementary material S4). For instance, a polygamous mating function can be produced by considering that a union is the pairing of 1 male and *h* females as per equation (4) of the main text (see Rosen (1983)).

As the “harmonic mean” function is unsuitable as a discrete-time mating function, one needs to turn to alternative solutions. Some studies use the “minimum function” (see, e.g., Jenouvrier et al. 2010), which ticks all the mandatory boxes but fails in the desirable properties 6b and 7. Focusing on one half of the problem (“*the number of pairs cannot exceed the number of females*” in Bessa-Gomes et al 2010); Legendre (2004) has developed the “modified harmonic mean” mating function: $U_{mh}\left( m,f \right)=\min\left( f,\frac{2mf}{m+f} \right)$, which corresponds to $U_{mh}^{*}\left( f^{*} \right)=\min\left( f^{*},2\left( 1-f^{*} \right)f^{*} \right)$ (plotted black with stars in Figure S1). The curve lays at the level of $U_{min,1}^{*}\left( f^{*} \right)$, which is the higher limit for any valid discrete-time mating function as per property 8, for $f^{*}<\frac{1}{2}$, but is above it for $f^{*}>\frac{1}{2}$. Therefore, this function - which was not designed, contrary to the aforementioned Hölder means function transferred to discrete-time models, as a monogamous function but as one clearly allowing polyandry, and as such has never been extended to polygynous models - does not suit the mandatory property 8 (also it does not obey desirable properties 6b and 7).

**References**

Bessa-Gomes, C., Legendre, S., & Clobert, J. (2010). Discrete two-sex models of population dynamics: on modelling the mating function. *Acta Oecologica*, *36*(5), 439-445.

Caswell, H. (2001). Matrix population models: construction, analysis, and interpretation. 2nd edn Sinauer Associates. *Inc., Sunderland, MA*.

Caswell, H., & Weeks, D. E. (1986). Two-sex models: chaos, extinction, and other dynamic consequences of sex. *The American Naturalist*, *128*(5), 707-735.

Fredrickson, A. G. (1971). A mathematical theory of age structure in sexual populations: random mating and monogamous marriage models. *Mathematical Biosciences*, *10*(1-2), 117-143.

Gupta, P. D. (1972). On two-sex models leading to stable populations. *Theoretical Population Biology*, *3*(3), 358-375.

Hadeler, K. P. (1989). Pair formation in age-structured populations. Acta Applicandae Mathematicae, 14(1–2), 91–102. <https://doi.org/10.1007/BF00046676>

Jenouvrier, S., Caswell, H., Barbraud, C., & Weimerskirch, H. (2010). Mating behavior, population growth, and the operational sex ratio: a periodic two-sex model approach. *The American Naturalist*, *175*(6), 739-752.

Legendre, S. (2004) ‘Age Structure, Mating System, and Population Viability’, in Evolutionary Conservation Biology. Cambridge University Press, pp. 41–58. doi: 10.1017/CBO9780511542022.005.

Lindström, J., & Kokko, H. (1998). Sexual reproduction and population dynamics: the role of polygyny and demographic sex differences. *Proceedings of the Royal Society of London. Series B: Biological Sciences*, *265*(1395), 483-488.

Miller, T. E. X., Shaw, A. K., Inouye, B. D., & Neubert, M. G. (2011). Sex-biased dispersal and the speed of two-sex invasions. American Naturalist, 177(5), 549–561. <https://doi.org/10.1086/659628>

Pollak, R. A. (1990). Two-sex demographic models. *Journal of Political Economy*, *98*(2), 399-420.

Pollak, R. A. (1986). A reformulation of the two-sex problem. *Demography*, *23*, 247-259.

Pollak, R. A. (1987). The two-sex problem with persistent unions: a generalization of the birth matrix-mating rule model. *Theoretical Population Biology*, *32*(2), 176-187.

Ranta, E., & Kaitala, V. (1999). Punishment of polygyny. *Proceedings of the Royal Society of London. Series B: Biological Sciences*, *266*(1435), 2337-2341.

Rosen, K. H. (1983). Mathematical models for polygamous mating systems. Mathematical Modelling, 4(1), 27–39. <https://doi.org/10.1016/0270-0255(83)90031-3>

Schoen, R. (1981). The harmonic mean as the basis of a realistic two-sex marriage model. *Demography*, *18*, 201-216.

Yellin, J., & Samuelson, P. A. (1974). A dynamical model for human population. *Proceedings of the National Academy of Sciences*, *71*(7), 2813-2817.

**Supplement 2:** The construction of the minharmonic mating function, and the computation of the mating efficiency

We construct the *minharmonic* mating function as a mixture of a minimum mating function and a modified harmonic mean function (corresponding to a harmonic mean mating function of parameter *a*, to be determined, with a non-zero intercept, *b*, to be determined). The mixing is performed according to a parameter *p* with $0\leq p\leq0.5$: for$f^{*}\leq p$ or $f^{*}\geq1-p$ (unbalanced OSRs), the *minharmonic* correspond to the “minimum” mating function of parameter 1 and for $p\leq f^{*}\leq1-p$ (balanced OSRs) to the harmonic mean function of parameter *a* and intercept *b*:

$$U^{*}\left( f^{*} \right)=\left\{ \begin{aligned} \min\left( f^{*}, 1-f^{*} \right)for f^{*}\leq p and f^{*}\geq1-p \\ af^{*}\left( 1-f^{*} \right)+b for p\leq f^{*}\leq1-p \end{aligned} \right. (10)$$

As we want this function to be continuous (see properties in Supplementary material S1), therefore we need $U^{*}\left( p^{-} \right)=U^{*}\left( p^{+} \right)$, which corresponds to $ap\left( 1-p \right)+b=min(p,1-p))=p$ and $\frac{dU^{*}}{df^{*}}\left( p^{-} \right)=\frac{dU^{*}}{df^{*}}\left( p^{+} \right)$, which corresponds to $a\left( 1-2p \right)=1$. These two equations lead to $a=\frac{1}{1-2p}$ and $b=p\left( 1-a\left( 1-p \right) \right)=\frac{-p^{2}}{1-2p}$, so that we can write the minharmonic function (equation (11)) as:

$$U_{minh,p}^{*}\left( f^{*} \right)=\left\{ \begin{aligned} \min\left( f^{*},1-f^{*} \right)for f^{*}\leq p and f^{*}\geq1-p \\ \frac{f^{*}\left( 1-f^{*} \right)-p^{2}}{1-2p} for p\leq f^{*}\leq1-p \end{aligned} \right.(11)$$

We define *e* as the mating efficiency at the balanced sex ratio: $e=E^{*}\left( 0.5 \right)=2*U^{*}\left( 0.5 \right)$. From equation (11), we get:

$e=2\times\frac{0.5\left( 1-0.5 \right)-p^{2}}{1-2p}=\frac{(0.5-p)(0.5+p)}{(0.5-p)}=0.5+p$.

We can therefore use parameter $e=0.5+p$, with $0.5\leq e\leq1$, instead of *p* to define the minharmonic mating function, as equation (11), where *p* is replaced by $e-0.5$ leads to equation (3) of the main text:

$$U_{minh,e}^{*}\left( f^{*} \right)=\left\{ \begin{aligned} \min\left( f^{*},1-f^{*} \right)for f^{*}\leq e-0.5 and f^{*}\geq1.5-e \\ \frac{f^{*}\left( 1-f^{*} \right)-{(e-0.5)}^{2}}{2(1-e)} for e-0.5\leq f^{*}\leq1.5-e \end{aligned} \right. (3)$$

For this mating function we have:

$$E_{minh,e}^{*}\left( f^{*} \right)=\left\{ \begin{aligned} 1 for f^{*}\leq e-0.5 and f^{*}\geq1.5-e \\ \frac{f^{*}\left( 1-f^{*} \right)-\left( e-0.5 \right)^{2}}{2\left( 1-e \right)\min\left( f^{*},1-f^{*} \right)} for e-0.5\leq f^{*}\leq1.5-e \end{aligned} \right. (12)$$

From which one can easily verify that$E_{minh,e}^{*}\left( 0.5 \right)=e$.

This defines the minharmonic function for all $0\leq p\leq0.5$, that is for all $0.5<e\leq1$, a segment which corresponds to harmonic mean functions of parameters $p>1$ for which they are flawed as discrete time monogamous mating functions (see Supplementary material S1). However, for a parameter $p\leq0.5,$that is $0\leq e<0.5$, the harmonic mean function is valid, which allows to extend our definition of the minharmonic function for all possible $e$ ($e$, being a probability, this is for all $0\leq e\leq1$): for $0\leq e<0.5$, the minharmonic mating function is simply the harmonic mean function of parameter *p=2e* (from $e=E_{h,p}^{*}\left( 0.5 \right)=0.5*p$), leading to $U_{minh,e}^{*}\left( f^{*} \right)=2ef^{*}\left( 1-f^{*} \right)$.

By construction, and by contrast to the harmonic mean function of parameter $p>1$ (because of property 8 of Supplementary material S1) and the minimum mating function (because of properties 6b and 7), the minharmonic function of parameter *e* obeys all the properties set forth in Supplementary material S1.

**Supplement S3.** Effect of harem size and mating efficiency on the component (minimum or modified harmonic) of the minharmonic used

The minharmonic mating function, for a parameter $0.5\leq e\leq1$, is built as a mixture of the minimum function of parameter *p=1* (for $0\leq f^{*}\leq e-0.5$ and, symmetrically, $0\leq1-f^{*}\leq e-0.5$) and a function corresponding to the harmonic mean function of parameter $p=\frac{1}{2(1-e)}$ with an intercept of $\frac{-{(e-0.5)}^{2}}{2(1-e)}$(for $e-0.5\leq f^{*}\leq1.5-e$) (see Supplementary material S2). For $0.5<e\leq1$, the latter therefore does not correspond to the harmonic mean function (of any parameter *p*). Only in the extreme case $e=0.5$, is the minharmonic function equal to the harmonic mean function (of parameter *p=1*) on its entire range.

On Figure S2, we represent the population growth rate and the operational sex ratio (OSR) for the wild boar population of the application (see main text) with the minharmonic function of parameter *e=0.9* for various value of *h* as per Figure 2 of the main text. We add some additional information, here, on the component of the mixture of the minharmonic mating function (dashed line when it corresponds to the minimum function and solid line when it corresponds to the modified harmonic mean function). In general, as per equation (5) from main text, the polygynous minharmonic function corresponds to the polygynous extension of the modified harmonic mean function (which is not a harmonic mean function, unless for $e\leq0.5$) when$(e-0.5)(f+mh)\leq f\leq(1.5-e)(f+mh)$ that is when $\frac{h(e-0.5)}{1+(e-0.5)(h-1)}\leq\frac{f}{f+m}=f^{*} \leq\frac{h(1.5-e)}{1+(1.5-e)(h-1)}$. Away from that interval, the polygynous minharmonic corresponds to the (polygynous) minimum function. For $e=0.9$, this interval corresponds to $\frac{0.4h}{1+0.4(h-1)}\leq f^{*} \leq\frac{0.6h}{1+0.6(h-1)}$, that is for $h=1$ to $0.4\leq f^{*} \leq0.6$and for $h=2$ to $0.5714\leq f^{*} \leq0.75$. The interval corresponding to the modified harmonic reduces as *h* and *e* increase and it shifts to the right as *h* increases. As a consequence, a given OSR will correspond to different components of the mixture as a function of *h*. For instance, for efficiency *e=0.9*, an OSR of 0.6 corresponds to the modified harmonic only for *h=2* (considering only integers for *h*).


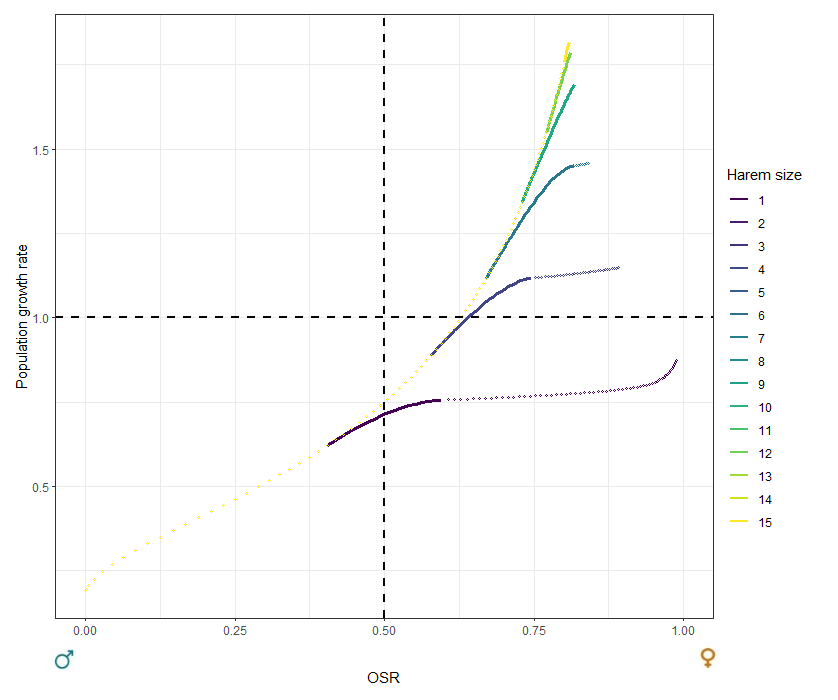


Figure S2. Population growth rate in relation to the OSR for different values of the harem size (indicated by colour, ranging from blue for low harem size to yellow for high harem size), in a wild boar population modelled with the minharmonic mating function *e=0.9*. Dashed lines (respectively solid lines) correspond to points where the minimum (respectively modified harmonic) component of the minharmonic function is used.

**Supplement 4.** Various mating systems and application to the minharmonic mating function.

1/ Mating systems

- *Polygynous mating system*

Polygyny corresponds to one male mating with *h* females, with *h* higher than 1. As per the application of the main text, our minharmonic mating function can be extended to polygynous mating system, via equation (4) (from the main text) such as:

$$U_{minh,e}^{pg}\left( m,f,h \right)=\left\{ \begin{aligned} \frac{1}{h}\min\left( f,mh \right)for \frac{f}{hm+f}\leq(e-0.5) and \frac{f}{hm+f}\geq(1.5-e) \\ \frac{\frac{mf}{(hm+f)}-\frac{(hm+f)}{h}{(e-0.5)}^{2}}{2(1-e)} for (e-0.5)\leq\frac{f}{hm+f}\leq(1.5-e) \end{aligned} (5) \right.$$

We can produce the monogamous version of it from equation (5) by setting $h=1$, which yields:

$$U_{minh,e}\left( m,f \right)=\left\{ \begin{aligned} \min\left( f,m \right)for \frac{f}{m+f}\leq(e-0.5) and \frac{f}{m+f}\geq(1.5-e) \\ \frac{\frac{mf}{(m+f)}-(m+f){(e-0.5)}^{2}}{2(1-e)} for (e-0.5)\leq\frac{f}{m+f}\leq(1.5-e) \end{aligned} (7) \right.$$

- *Polyandrous mating system*

Polyandry corresponds to one female mating with *h’* males, with *h’* higher than 1. Any correctly built mating function can easily be extended to a polyandrous framework (as polygynous framework in the main text), with harems of males of mean size *h’*, such as:

$$U^{pa}\left( m,f,h' \right)=U\left( \frac{m}{h'},f \right)=\left( f+\frac{m}{h^{'}} \right)U^{*}\left( \frac{h^{'}f}{m+h^{'}f} \right) (13)$$

Where $U^{pa}\left( m,f,h' \right)$ is the number of harems formed. For the minharmonic function, this leads to:

$U_{minh,e}^{pa}\left( m,f,h' \right)=\left\{ \begin{aligned} \frac{1}{h'}\min\left( fh',m \right)for \frac{h^{'}f}{m+h^{'}f}\leq\left( e-0.5 \right) and \frac{h^{'}f}{m+h^{'}f}\geq(1.5-e) \\ \frac{\frac{mf}{(m+fh')} - \frac{(m+fh')}{h'}{(e-0.5)}^{2}}{2(1-e)} for (e-0.5)\leq\frac{h^{'}f}{m+h^{'}f}\leq(1.5-e) \end{aligned} \right.$ (14)

- *Promiscuous mating system*

One can also extend a (monogamous) mating function to a promiscuous mating system, where *h’* males share a harem of *h* females, via:

$$U^{pro}\left( m,f,h,h' \right)=U\left( \frac{m}{h^{'}},\frac{f}{h} \right)=\frac{1}{hh'}\left( h^{'}f+hm \right)U^{*}\left( \frac{h'f}{hm+h'f} \right) (15)$$

Where $U^{pro}\left( m,f,h,h' \right)$ is the number of unions (i.e. reproductive groups made of $h'$ males and $h$ females) formed. If $h=h'=1$ , it corresponds to the monogamous case, if $h'=1$ to the polygynous case and if $h=1$ to the polyandrous case.

Our function in the case of promiscuity can be written as:

$U_{minh,e}^{pro}\left( m,f \right)=\left\{ \begin{aligned} \frac{1}{hh'}\min\left( fh^{'},mh \right)for \frac{h'f}{hm+h'f}\leq e-0.5 and \frac{h'f}{hm+h'f}\geq1.5-e \\ \\ \frac{\frac{mf}{(mh+fh^{'})} - \frac{fh^{'}+mh}{hh'}{(e-0.5)}^{2}}{2(1-e)} for e-0.5\leq\frac{h'f}{hm+h'f}\leq1.5-e \\ \end{aligned} \right.$(16)

2/ Birth functions

From a union function, one can compute the birth function $Bf$ ($Bm$ for a male, respectively), corresponding to the expected number of offspring produced by an adult female (an adult male, respectively) taken at random in the population.

This is $Bf=Pr(f)*K$ where $Pr(f)=\frac{hU}{f}$ is the probability to be in a union and $K$ the number of offspring produced by a female in a union:

$$Bf=\frac{hKU}{f} (17)$$

For males, we have similarly $Bm=Pr(m)*K_{m}$ where $Pr(m)=\frac{h'U}{m}$ is the probability to be in a union and $K_{m}$ the number of offspring produced by a male in a union. Overall, a union yields $h^{'}K_{m}=hK$ offspring so that $K_{m}=\frac{h}{h^{'}}K$, leading to:

$Bm=\frac{hKU}{m} (18)$

These two equations can be reached directly by considering that the total number of offspring produced in a season at the population level is $hKU$ ($hK$ offspring produced by a union formed).

**Supplement 5.** Building two-sex body size-structured population matrix for the wild boar population of Châteauvillain, France

We built an average standard 6×6 two-sex body size-structured matrix **A** (Table 1 of the main text) that included mean annual probabilities (estimated in Gamelon et al*.* 2012, see Table 2 in main text) for an individual to grow up to an upper class, survival probabilities (i.e. the product of natural and hunting survival) and reproduction. We built a pre-breeding census model. To construct **A**, we considered three different phases. The population vector corresponds to, in that order: small females, medium females, large females, small males, medium males and large males.

The first phase occurs in spring, when only medium and large individuals (females and males) reproduce. Reproduction is modelled by fertility matrix **F**.

$$\mathbf{F}=0.5\times s0\times\left[ \begin{matrix} 0 & 0.5\times{Bf}_{M} & 0.5\times{Bf}_{L} & 0 & 0.5\times Bm & 0.5\times Bm \\ 0 & 0 & 0 & 0 & 0 & 0 \\ 0 & 0 & 0 & 0 & 0 & 0 \\ 0 & 0.5\times{Bf}_{M} & 0.5\times{Bf}_{L} & 0 & 0.5\times Bm & 0.5\times Bm \\ 0 & 0 & 0 & 0 & 0 & 0 \\ 0 & 0 & 0 & 0 & 0 & 0 \end{matrix} \right]\left( 19 \right)$$

We assumed a balanced sex ratio at birth (Servanty et al. 2007); hence the factor 0.5 applied to each element of the matrix of equation (19). Individuals of a given sex and in a given size class produce offspring according to their birth function (see Supplementary material S6). Small individuals do not mate. Medium and large males have the same birth function $Bm$; ${Bf}_{M}$ is the birth function for medium females and ${Bf}_{L}$ that of large females. An offspring is produced by both a male and a female, therefore we have a factor 0.5 in front of equation (19). Further the fertility matrix accounts for postnatal survival of offspring (via $s0$).

From a pre-breeding population vector $\boldsymbol{n,}$the population after the first reproductive phase is $(\boldsymbol{I+F})\boldsymbol{n}$**,** where ***I*** is the identity matrix, modelling the surviving individuals (they all survive this phase, and survival is accounted for in the third phase described below).

The reproductive phase is followed by a phase of growth: growth rates differ according for newborn (or juveniles) and non-newborn (adults). For juvenile wild boars, the probability to reach the small or medium size-class during their first year is modelled by growth matrix of juveniles **G**_juveniles_. Adult wild boars either grow to an upper size-class or stay in their size class; and this is modelled by the growth matrix of adults **G**_adults_**_._** In that matrix $G_{Xy}$is the probability for an adult of sex *y* and size *X-1* to grow towards size *X* and therefore (1-$G_{Xy}$) the probability for it to remain in the same size *X-1* during that year. Class *L* is the largest size class, therefore for large individuals, the probability to remain in that class is 1. The mean annual growth probabilities are estimated in Gamelon *et al.* (2012) (see Table 2 of the main text).

$$\boldsymbol{G}_{juveniles}=\left[ \begin{matrix} {1-G}_{juvMf} & 0 & 0 & 0 & 0 & 0 \\ G_{juvMf} & 0 & 0 & 0 & 0 & 0 \\ 0 & 0 & 0 & 0 & 0 & 0 \\ 0 & 0 & 0 & {1-G}_{juvMm} & 0 & 0 \\ 0 & 0 & 0 & G_{juvMm} & 0 & 0 \\ 0 & 0 & 0 & 0 & 0 & 0 \end{matrix} \right] (20a)$$

$$\boldsymbol{G}_{adults}=\left[ \begin{matrix} (1-G_{Mf}) & 0 & 0 & 0 & 0 & 0 \\ G_{Mf} & (1-G_{Lf}) & 0 & 0 & 0 & 0 \\ 0 & G_{Lf} & 1 & 0 & 0 & 0 \\ 0 & 0 & 0 & (1-G_{Mm}) & 0 & 0 \\ 0 & 0 & 0 & G_{Mm} & (1-G_{Lm}) & 0 \\ 0 & 0 & 0 & 0 & G_{Lm} & 1 \end{matrix} \right]\left( 20b \right)$$

Because of the differentiation between juveniles and adults, the two different sets of growth rates apply to different components of the population: $\boldsymbol{G}_{adults}$ apply to the “survivors” of the reproductive phase and $\boldsymbol{G}_{juveniles}$ to the newborn. Therefore, from a pre-breeding population vector $\boldsymbol{n,}$the population vector, after the first two phases, reproduction, and growth, is equal to $(\boldsymbol{G}_{adults}\boldsymbol{\times I+}\boldsymbol{G}_{juveniles}\boldsymbol{\times F})\boldsymbol{n}$**.**

Finally, the third and last phase correspond to survival. Wild boar individuals encounter both natural death and death caused by hunting in their new size class (see Gamelon et al. 2012). The survival matrix **S** provides, for each size class and sex, the annual survival factoring both natural and hunting-caused death. The mean natural and hunting survival probabilities over the studied period were estimated by Gamelon *et al.* (2012) for each sex and size class (see Table 2 in the main text). Here $SXy$ is the annual survival probability (probability not to die of natural death) for an individual of sex *y* and size *X* and $HXy$ is the probability to be shot by hunters for the same individual (for medium and large females, the probability to be killed is the same and denotes *Hf*).

$$\boldsymbol{S}=\left[ \begin{matrix} SSf\times(1-HSf) & 0 & 0 & 0 & 0 & 0 \\ 0 & SMf\times(1-Hf) & 0 & 0 & 0 & 0 \\ 0 & 0 & SLf\times(1-Hf) & 0 & 0 & 0 \\ 0 & 0 & 0 & SSm\times(1-HSm) & 0 & 0 \\ 0 & 0 & 0 & 0 & SMm\times(1-HMm) & 0 \\ 0 & 0 & 0 & 0 & 0 & SLm\times(1-HLm) \end{matrix} \right]\left( 21 \right)$$

Overall, from a pre-breeding census population vector $\boldsymbol{n}_{t}\boldsymbol{,}$the population after the three phases making up the year $\boldsymbol{n}_{t+1}=\mathbf{S}\left( \boldsymbol{G}_{adults}\boldsymbol{\times I+}\boldsymbol{G}_{juveniles}\boldsymbol{\times F} \right)\boldsymbol{n}_{t}$**.** In other words, the annual dynamics of the population consists of the sequence of these three phases and is therefore modelled by:

$$\boldsymbol{A=} \boldsymbol{S}\left( \boldsymbol{G}_{adults}+\boldsymbol{G}_{juveniles}\boldsymbol{F} \right)\left( 22 \right).$$

Which we present in matrix form as Table 1 of the main text.

**References**

Gamelon, M., Gaillard, J. M., Servanty, S., Gimenez, O., Toïgo, C., Baubet, E., ... & Lebreton, J. D. (2012). Making use of harvest information to examine alternative management scenarios: a body weight‐structured model for wild boar. *Journal of Applied Ecology*, *49*(4), 833-841.

Servanty, S., Gaillard, J. M., Allainé, D., Brandt, S., & Baubet, E. (2007). Litter size and fetal sex ratio adjustment in a highly polytocous species: the wild boar. *Behavioral Ecology*, *18*(2), 427-432.

**Supplement 6.** Size-structured birth functions for the wild boar population at Châteauvillain, France

Birth functions for females (*Bf*) and for males (*Bm*) provide the expected number of offspring produced by a female and a male, respectively. It corresponds to the expected number of offspring produced by an individual (of a given sex) in the population taken at random (in a union or not).

In a population structured by sex only and monogamous, it is $Bf=\Pr\left( f \right)\times K$and $Bm=\Pr\left( m \right)\times K$ where $\Pr\left( f \right)$($\Pr\left( m \right)$, respectively) is the probability for a female (a male, respectively) to be in a union; with $\Pr\left( f \right)=\frac{U(m,f)}{f}$ , $\Pr\left( m \right)=\frac{U(m,f)}{m}$ and *K*, the mean number of offspring produced by a union, that is by a mated female (see Supplementary material S4).

In a population structured by sex only and polygynous with a mean harem size *h*, we still have $Bf=\Pr\left( f \right)\times K$ but $Bm=\Pr\left( m \right)\times K\times h$ as a mated male produces *K* offspring per female in its harem. Further the probability for a male to be in a union (that is, a harem) is still $\Pr\left( m \right)=\frac{U(m,f)}{m}$ but the probability for a female to be in a union is now $\Pr\left( f \right)=h\frac{U(m,f)}{f}$ as there are *h* females per union. Put together, and focusing on the minharmonic mating function, this yields to the equations (6a) and (6b) in the main text:

$Bf=\Pr\left( f \right)\times K=h\frac{U_{minh,e}^{pg}\left( m,f,h \right)}{f}\times K=K\times h\times\frac{U_{minh,e}^{pg}\left( m,f,h \right)}{f}$ and

$Bm=K\times h\times\frac{U_{minh,e}^{pg}\left( m,f,h \right)}{m}$; as per Supplementary material S4.

In the application of the main text, these functions are size-structured due to a difference in the number of offspring produced by females of different body-size classes. Medium females produce less offspring (i.e. *K_M_* = 5 offspring per litter; Gamelon et al. 2012) than large females (i.e. *K_L_* = 6 offspring per litter; Gamelon et al. 2012). In this case, we built two birth functions for females: ${Bf}_{M}$ for medium females and ${Bf}_{L}$ for large females. We consider that $\Pr\left( f_{M} \right)=\Pr\left( f_{L} \right)=\Pr\left( f \right),$ is that all females have the same probability to enter a harem, independently from body-size and therefore from (6a) we get:

${Bf}_{M}=K_{M}\times h\times\frac{U_{minh,e}^{pg}(m,f,h)}{f}$

${Bf}_{L}=K_{L}\times h\times\frac{U_{minh,e}^{pg}(m,f,h)}{f}$

Similarly we consider that $\Pr\left( m_{M} \right)=\Pr\left( m_{L} \right)=\Pr\left( m \right)$, so that, from (6b), we have $Bm_{M}=Bm_{L}=Bm=K_{m}\times h\times\frac{U_{minh,e}^{pg}\left( m,f,h \right)}{m},$ where $K_{m}$is the mean number of offspring produced by a male, that is, by a harem. Because $\Pr\left( f_{M} \right)=\Pr\left( f_{L} \right)$, this is $K_{m}=\frac{f_{M}K_{M}+f_{L}K_{L}}{f}$.

Overall, we get: $Bm=\frac{f_{M}K_{M}+f_{L}K_{L}}{f}\times h\times\frac{U_{minh,e}^{pg}(m,f,h)}{m}$.

**References**

Gamelon, M., Gaillard, J. M., Servanty, S., Gimenez, O., Toïgo, C., Baubet, E., ... & Lebreton, J. D. (2012). Making use of harvest information to examine alternative management scenarios: a body weight‐structured model for wild boar. *Journal of Applied Ecology*, *49*(4), 833-841.

**Supplement 7.** Relationship between the proportion of breeding females killed by hunters *Hf*, the operational sex ratio, and the population growth rate.

The operational sex ratio (OSR) and the population growth rate both depend on the proportion of breeding females killed by hunting (*Hf*). In Figures S3-1 and S3-2, considering as a mating function the minharmonic of efficiency *e=0.8*, we display, as a function of *Hf*, the OSR and the population growth rate at the equilibrium (the four 3D plots display the same output, but considered from four different angles). Considering only the OSR and the population growth rate, these 3D plot corresponds to the 2D plot at the bottom left of Figure 2 (main text). Figures S3-1 and S3-2 show that an increase of the proportion of breeding females killed (increase in *Hf*) leads to a decrease in the OSR, and therefore in the population growth rate. For instance, for a monogamous mating system (Figure S3-1), the increase of the proportion of breeding females killed from 0.01 to 0.99, led the OSR to decrease from 0.99 to 0, corresponding population growth rates of 0.87 and 0.19, respectively. In a polygynous mating system with a harem size of *h=15* (Figure S3-2), the same increase in the proportion of females killed led the OSR to decrease from 0.80 to 0, and the corresponding population growth rate to decrease from 1.8 to 0.19.

In the exploited population of Châteauvillain-Arc-en-Barrois (France), the proportion of breeding females killed is *Hf*=0.51, however, we ignore the efficiency in mating and the harem size in this population. In absence of reliable estimates of harem size and mating efficiency in this wild boar population, we considered the current OSR from a fixed mating efficiency (*e=0.8*) and a harem size varying from 1 (i.e. monogamy) to 15 (i.e. polygyny), leading to ${OSR}_{Châteauvillain}=0.67$ for a monogamous mating system and to 0.63 for polygynous mating system with *h=15*. The corresponding population growth rate is 0.76
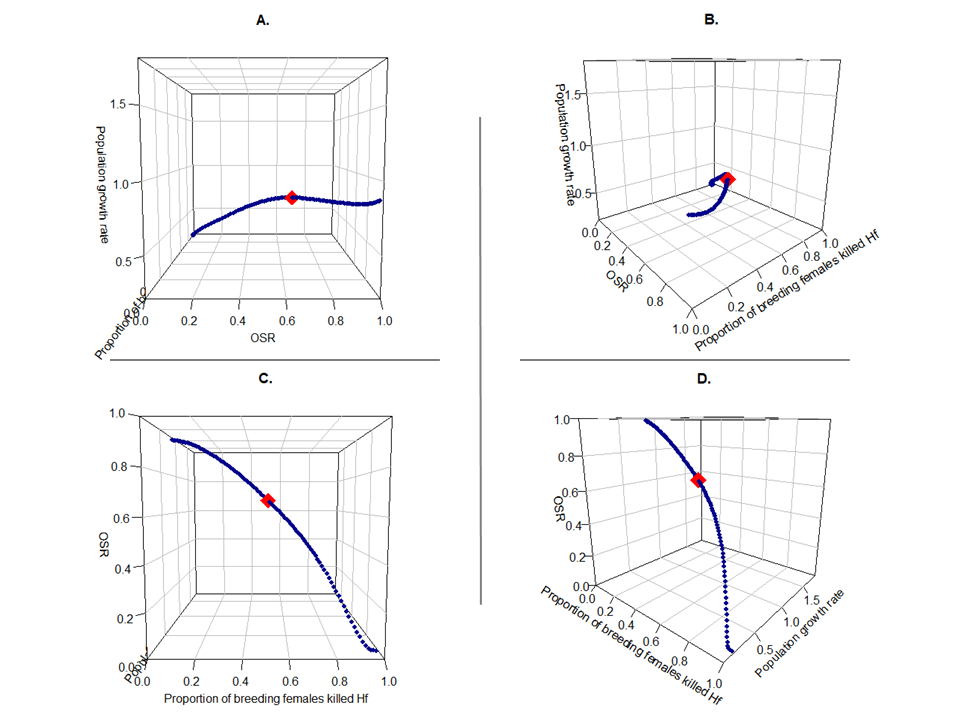
for monogamy and 1.00 for polygyny (see red diamonds on the Figures S3-1 and S3-2).

Figure S3-1. The relation between the proportion of breeding females killed by hunting (*Hf*), the OSR, and the population growth rate as a 3D plot seen from four different angles (A. B. C. and D.), for the harem size *h=1* (i.e. monogamy) and a mating efficiency of 0.8. The red diamonds correspond to the current proportion of breeding killed females *Hf* of the exploited population of wild boars from Châteauvillain.


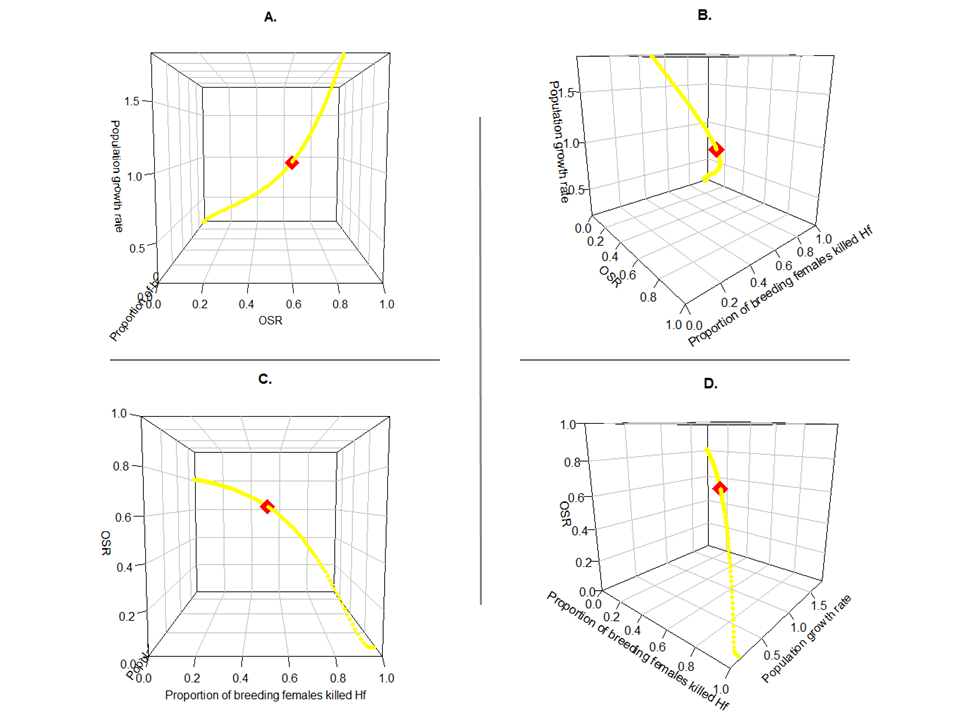


Figure S3-2. The relation between the proportion of breeding females killed by hunting (*Hf*), the OSR, and the population growth rate as a 3D plot seen from four different angles (A. B. C. and D.), for the harem size *h=15* and a mating efficiency of 0.8. The red diamonds correspond to the current proportion of breeding killed females *Hf* of the exploited population of wild boars from Châteauvillain.
